# Supplementary figures and images for: LncRNA HOTAIR promotes proliferation, invasion and migration in NSCLC cells via the CCL22 signaling pathway
Source: PLoS One. 2022 Feb 17;17(2):e0263997. doi: 10.1371/journal.pone.0263997 (PMC8853541; doi:10.1371/journal.pone.0263997)

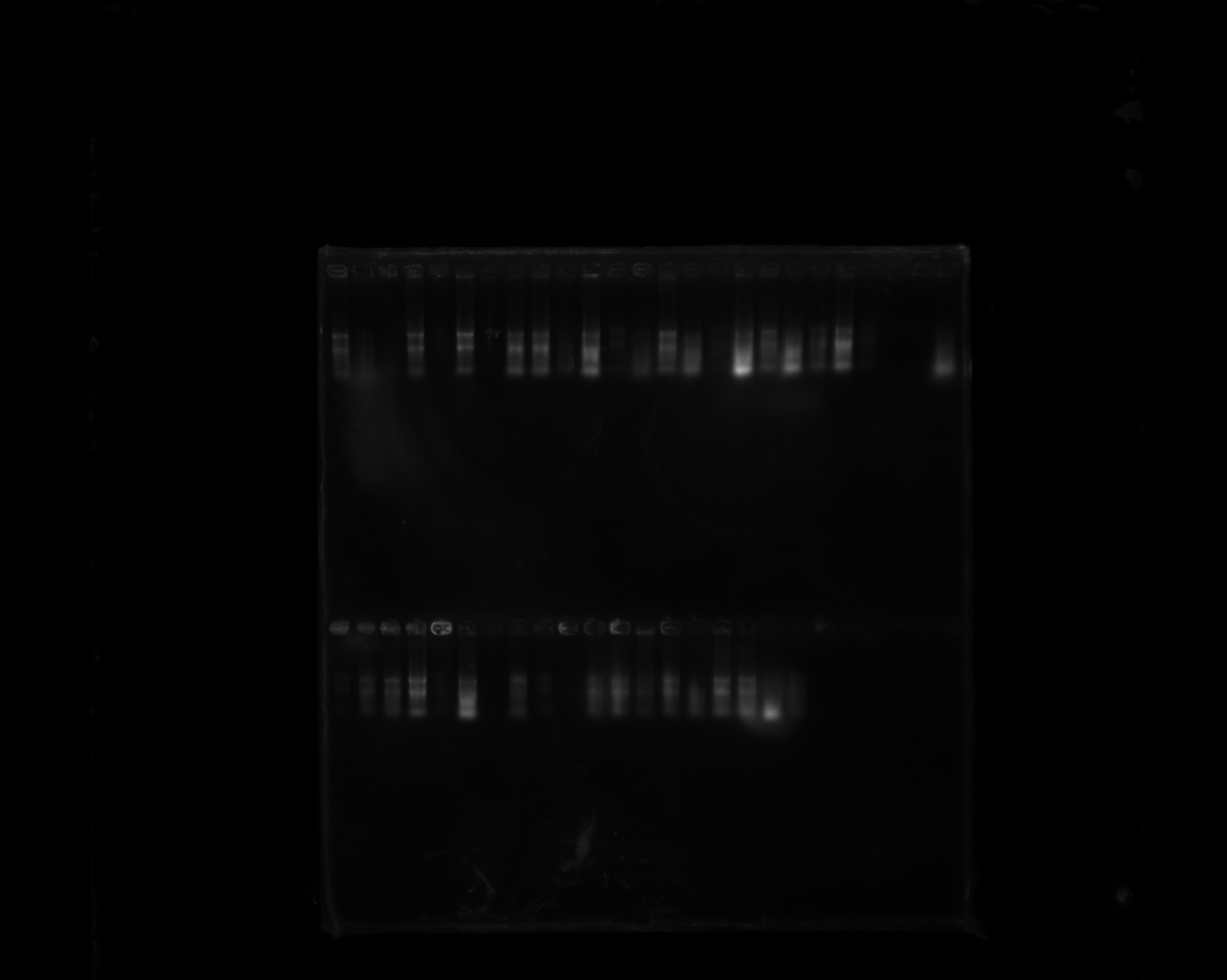

Supplement: S1 Data — (ZIP) [file pone.0263997.s001.zip › original date(excluding IHC images)/original data-figure1/A B QPCR/RNA-Gel electrophoresis.tif]

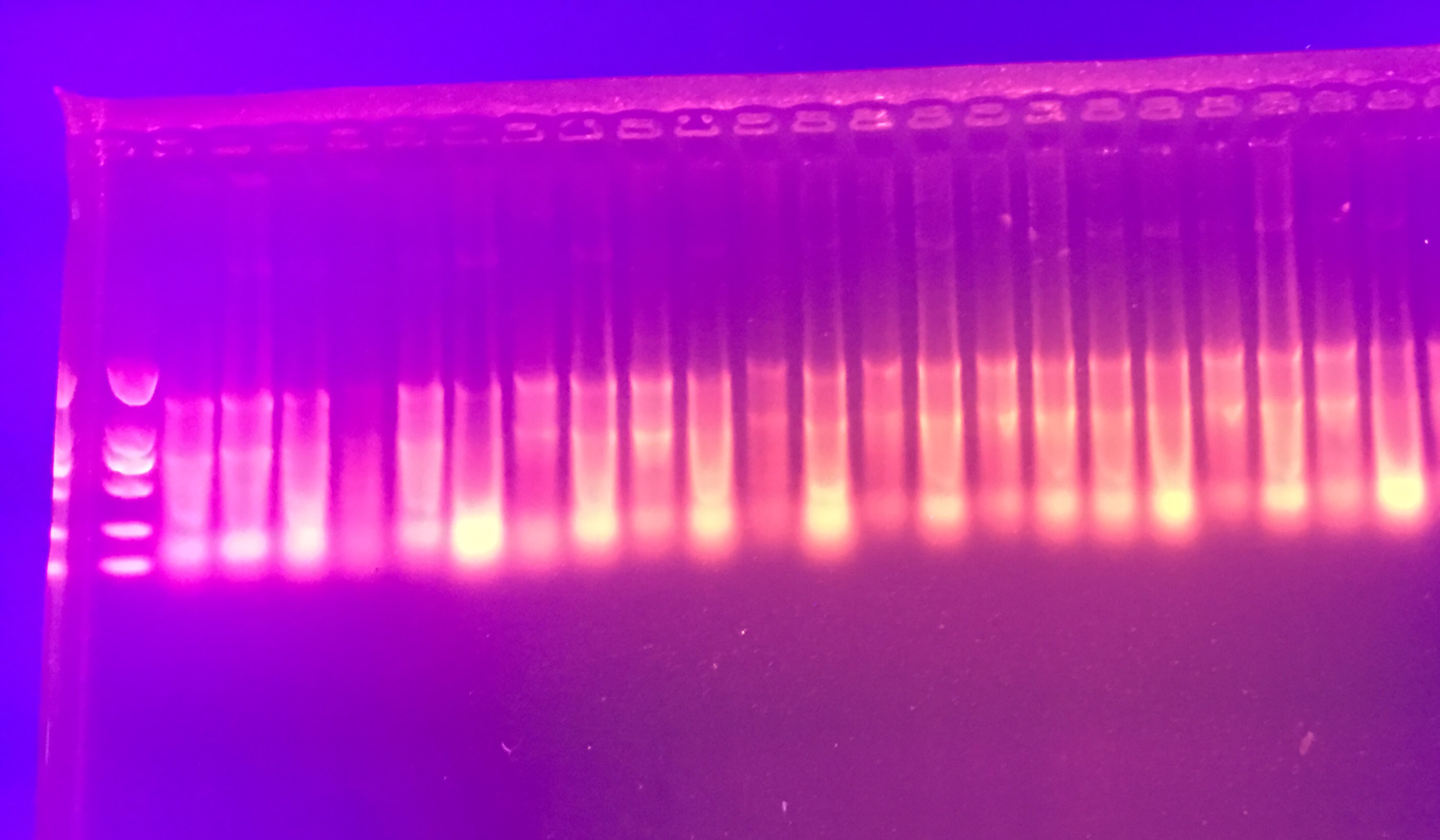

Supplement: S1 Data — (ZIP) [file pone.0263997.s001.zip › original date(excluding IHC images)/original data-figure1/A B QPCR/tissues-Gel electrophoresis.jpg]

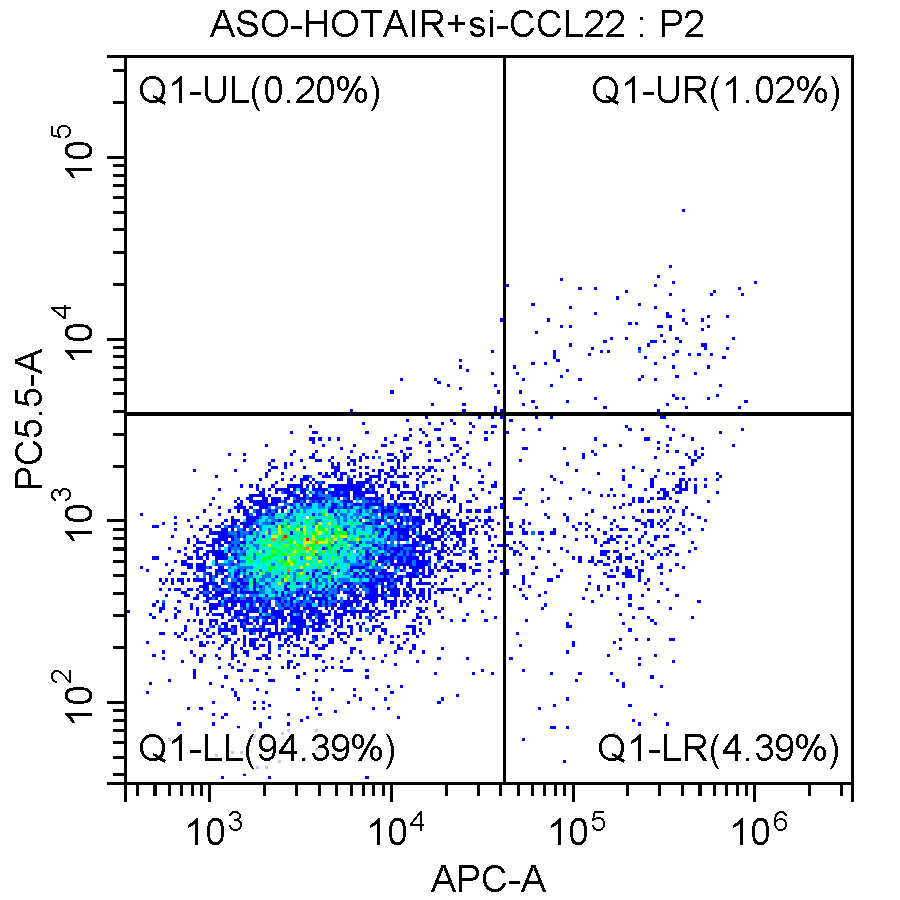

Supplement: S1 Data — (ZIP) [file pone.0263997.s001.zip › original date(excluding IHC images)/original data-figure3/B Apoptosis/ASO-HOTAIR+si-CCL22_Plot1.bmp]

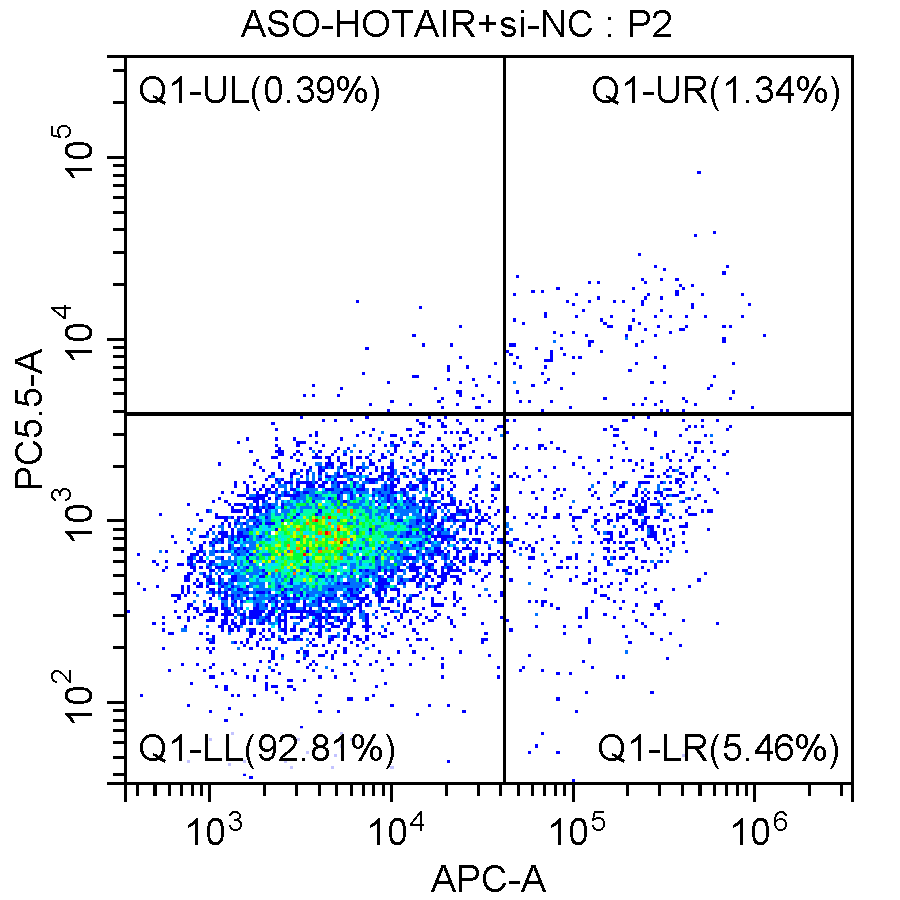

Supplement: S1 Data — (ZIP) [file pone.0263997.s001.zip › original date(excluding IHC images)/original data-figure3/B Apoptosis/ASO-HOTAIR+si-NC_Plot1.bmp]

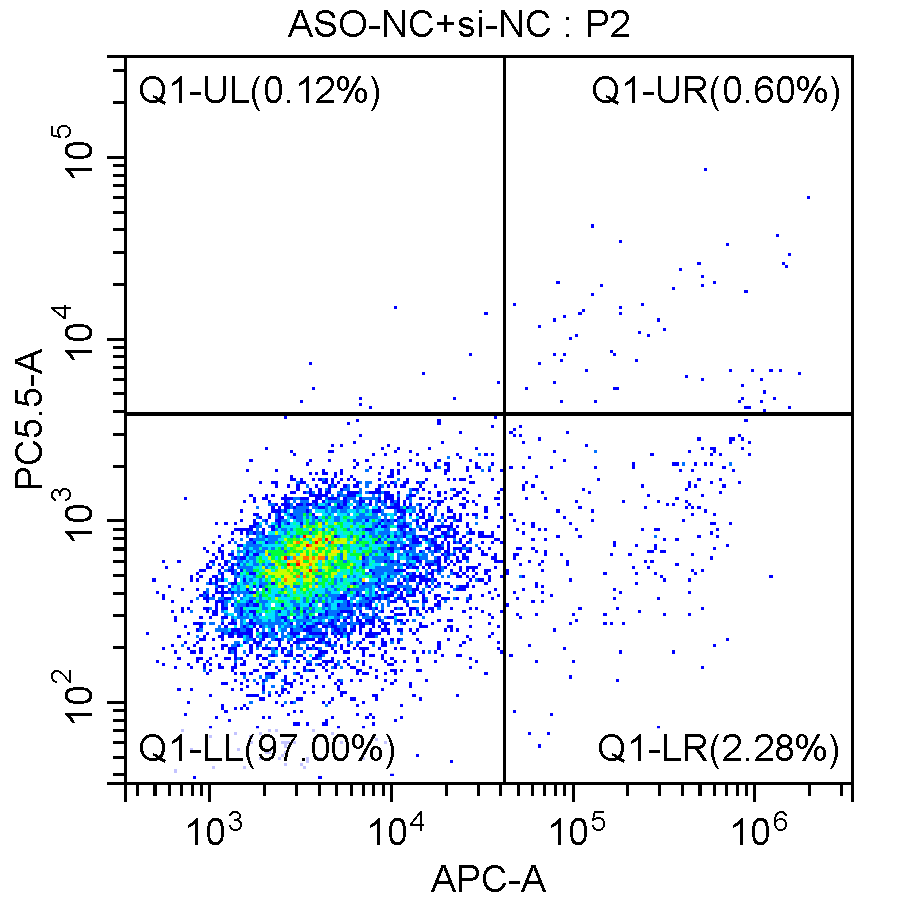

Supplement: S1 Data — (ZIP) [file pone.0263997.s001.zip › original date(excluding IHC images)/original data-figure3/B Apoptosis/ASO-NC+si-NC_Plot1.bmp]
