# Supplementary material for: LncRNA HOTAIR promotes proliferation, invasion and migration in NSCLC cells via the CCL22 signaling pathway
Source: PLoS One. 2022 Feb 17;17(2):e0263997. doi: 10.1371/journal.pone.0263997 (PMC8853541; doi:10.1371/journal.pone.0263997)
Supplement: Attachment — Submitted filename: Response to Reviewers.doc [file pone.0263997.s002.doc]

**Dear Editor and reviewer,**

**Thank you for your valuable advice. We have made changes one by one according to the comments. Our response is as follows！**

PONE-D-21-21513

Exploring the mechanisms of the immune regulation by LncRNA HOTAIR and CCL22 signaling pathways about non-small cell lung cancer

PLOS ONE

Dear Dr. Liang,

Thank you for submitting your manuscript to PLOS ONE. After careful consideration, we feel that it has merit but does not fully meet PLOS ONE’s publication criteria as it currently stands. Therefore, we invite you to submit a revised version of the manuscript that addresses the points raised during the review process.

Please submit your revised manuscript by February 9,2022. If you will need more time than this to complete your revisions, please reply to this message or contact the journal office at [plosone@plos.org](mailto:plosone@plos.org). When you're ready to submit your revision, log on to <https://www.editorialmanager.com/pone/> and select the 'Submissions Needing Revision' folder to locate your manuscript file.

Please include the following items when submitting your revised manuscript:

- A rebuttal letter that responds to each point raised by the academic editor and reviewer(s). You should upload this letter as a separate file labeled 'Response to Reviewers'.
- A marked-up copy of your manuscript that highlights changes made to the original version. You should upload this as a separate file labeled 'Revised Manuscript with Track Changes'.
- An unmarked version of your revised paper without tracked changes. You should upload this as a separate file labeled 'Manuscript'.

If you would like to make changes to your financial disclosure, please include your updated statement in your cover letter. Guidelines for resubmitting your figure files are available below the reviewer comments at the end of this letter.

If applicable, we recommend that you deposit your laboratory protocols in [protocols.io](http://protocols.io/) to enhance the reproducibility of your results. Protocols.io assigns your protocol its own identifier (DOI) so that it can be cited independently in the future. For instructions see: [https://journals.plos.org/plosone/s/submission-guidelines#loc-laboratory-protocols](https://journals.plos.org/plosone/s/submission-guidelines#loc-laboratory-protocols). Additionally, PLOS ONE offers an option for publishing peer-reviewed Lab Protocol articles, which describe protocols hosted on [protocols.io](http://protocols.io/). Read more information on sharing protocols at <https://plos.org/protocols?utm_medium=editorial-email&utm_source=authorletters&utm_campaign=protocols>.

We look forward to receiving your revised manuscript.

Kind regards,

Suhwan Chang
Academic Editor
PLOS ONE

Journal Requirements:

When submitting your revision, we need you to address these additional requirements.

1. Please ensure that your manuscript meets PLOS ONE's style requirements, including those for file naming. The PLOS ONE style templates can be found at
<https://journals.plos.org/plosone/s/file?id=wjVg/PLOSOne_formatting_sample_main_body.pdf> and
<https://journals.plos.org/plosone/s/file?id=ba62/PLOSOne_formatting_sample_title_authors_affiliations.pdf>

2. In your Methods section, please provide additional details regarding the cell lines used in your study and ensure you have described the source. For more information regarding PLOS' policy on materials sharing and reporting, see [https://journals.plos.org/plosone/s/materials-and-software-sharing#loc-sharing-materials](https://journals.plos.org/plosone/s/materials-and-software-sharing#loc-sharing-materials), and for more information on PLOS ONE's guidelines for research using cell lines, see [https://journals.plos.org/plosone/s/submission-guidelines#loc-cell-lines](https://journals.plos.org/plosone/s/submission-guidelines#loc-cell-lines).

3. Your ethics statement should only appear in the Methods section of your manuscript. If your ethics statement is written in any section besides the Methods, please move it to the Methods section and delete it from any other section. Please ensure that your ethics statement is included in your manuscript, as the ethics statement entered into the online submission form will not be published alongside your manuscript.

4. Please remove all personal information, ensure that the data shared are in accordance with participant consent, and re-upload a fully anonymized data set.

Note: spreadsheet columns with personal information must be removed and not hidden as all hidden columns will appear in the published file.

Additional guidance on preparing raw data for publication can be found in our Data Policy ([https://journals.plos.org/plosone/s/data-availability#loc-human-research-participant-data-and-other-sensitive-data](https://journals.plos.org/plosone/s/data-availability#loc-human-research-participant-data-and-other-sensitive-data)) and in the following article: <http://www.bmj.com/content/340/bmj.c181.long>.

5. In your Data Availability statement, you have not specified where the minimal data set underlying the results described in your manuscript can be found. PLOS defines a study's minimal data set as the underlying data used to reach the conclusions drawn in the manuscript and any additional data required to replicate the reported study findings in their entirety. All PLOS journals require that the minimal data set be made fully available. For more information about our data policy, please see <http://journals.plos.org/plosone/s/data-availability>.

Upon re-submitting your revised manuscript, please upload your study’s minimal underlying data set as either Supporting Information files or to a stable, public repository and include the relevant URLs, DOIs, or accession numbers within your revised cover letter. For a list of acceptable repositories, please see [http://journals.plos.org/plosone/s/data-availability#loc-recommended-repositories](http://journals.plos.org/plosone/s/data-availability#loc-recommended-repositories). Any potentially identifying patient information must be fully anonymized.

Important: If there are ethical or legal restrictions to sharing your data publicly, please explain these restrictions in detail. Please see our guidelines for more information on what we consider unacceptable restrictions to publicly sharing data: [http://journals.plos.org/plosone/s/data-availability#loc-unacceptable-data-access-restrictions](http://journals.plos.org/plosone/s/data-availability#loc-unacceptable-data-access-restrictions). Note that it is not acceptable for the authors to be the sole named individuals responsible for ensuring data access.

We will update your Data Availability statement to reflect the information you provide in your cover letter.

6. Please review your reference list to ensure that it is complete and correct. If you have cited papers that have been retracted, please include the rationale for doing so in the manuscript text, or remove these references and replace them with relevant current references. Any changes to the reference list should be mentioned in the rebuttal letter that accompanies your revised manuscript. If you need to cite a retracted article, indicate the article’s retracted status in the References list and also include a citation and full reference for the retraction notice.

7. Please include captions for your Supporting Information files at the end of your manuscript, and update any in-text citations to match accordingly. Please see our Supporting Information guidelines for more information: <http://journals.plos.org/plosone/s/supporting-information>.

[Note: HTML markup is below. Please do not edit.]

Reviewers' comments:

Reviewer's Responses to Questions

**Comments to the Author**

1. Is the manuscript technically sound, and do the data support the conclusions?

The manuscript must describe a technically sound piece of scientific research with data that supports the conclusions. Experiments must have been conducted rigorously, with appropriate controls, replication, and sample sizes. The conclusions must be drawn appropriately based on the data presented.

Reviewer #1: Partly

Reviewer #2: Yes

2. Has the statistical analysis been performed appropriately and rigorously?

Reviewer #1: Yes

Reviewer #2: Yes

3. Have the authors made all data underlying the findings in their manuscript fully available?

The [PLOS Data policy](http://www.plosone.org/static/policies.action#sharing) requires authors to make all data underlying the findings described in their manuscript fully available without restriction, with rare exception (please refer to the Data Availability Statement in the manuscript PDF file). The data should be provided as part of the manuscript or its supporting information, or deposited to a public repository. For example, in addition to summary statistics, the data points behind means, medians and variance measures should be available. If there are restrictions on publicly sharing data—e.g. participant privacy or use of data from a third party—those must be specified.

Reviewer #1: Yes

Reviewer #2: Yes

4. Is the manuscript presented in an intelligible fashion and written in standard English?

PLOS ONE does not copyedit accepted manuscripts, so the language in submitted articles must be clear, correct, and unambiguous. Any typographical or grammatical errors should be corrected at revision, so please note any specific errors here.

Reviewer #1: No

Reviewer #2: Yes

5. Review Comments to the Author

Please use the space provided to explain your answers to the questions above. You may also include additional comments for the author, including concerns about dual publication, research ethics, or publication ethics. (Please upload your review as an attachment if it exceeds 20,000 characters)

Reviewer #1: Reviewer comments

The authors aimed to explore the mechanism of immune regulation of the long noncoding RNA and CCL22 and their role in the proliferation, migration, and invasion of non-small cell lung cancer. They build upon the existing knowledge and roles of these LncRNA and CCL22 by investigating their expression patterns as well as their effect on apoptosis and migration of NSCLC. Although their experimental procedures used for the various assays are effective, there are a few concerns about the organization of the manuscript

Concerns:
Materials and methods;

The oligonucleotides used in the study and their descriptions should be presented in a tabular form.

**Reply: We've tabulated primer sequences (Table 1).**
The procedure for transfection and maintenance of the cell cultures should be provided in this section.

**Reply: We have added this sections (Page 5, line 100-113).**
The experimental procedures for immunohistochemistry, qPCR, and flow cytometry must be detailed in different paragraphs or sub-sections.

**Reply: We have refined the method again (Page 6-8, line 115-163).**
The section needs to be revised grammatically and formatted according to the journal's submission requirements to make it an easier read.

**Reply: We have asked a professional English teacher to proofread the manuscript in the language. The format of the manuscript was also modified according to the journal.**

Results:

Although the figures and statical analysis are provided in the supporting documents, the authors can state the P-values with the statistical significance on the figures.

**Reply: We have re-marked the P value in the manuscript.**
Figure 3A can be labeled as 3Ai and 3Aii instead of left and right in the results section.

**Reply: We have restated the results (Page 9-10, line 186-192). Thank you for your comments!**
The font theme and font sizes are not uniform in the manuscript.

**Reply: They were done.**
The results section is poorly written and undermines the quality of the figures provided in the manuscripts. The results section should be revised.

**Reply: We have restated the result sections. Thank you for your comments.**

Minor concerns:
There are numerous grammatical errors in the manuscript that require revisions to make the manuscript easier to read.

**Reply: We have invited professional teachers to polish the manuscript.**

Reviewer #2: The manuscript entiltled "Exploring the mechanisms of the immune regulation by LncRNA HOTAIR and CCL22
signaling pathways about non-small cell lung cancer" are well documented. The results are clear abnd well presented.
materials and method are well elaborated.
The following issues need to be addressed.
1. The English need to be improved throughout the manuscript.

**Reply: We have invited professional teachers to polish the manuscript.**

2. There are several typing erros. please revise the whole manuscript.

**Reply: We have asked the full manuscript to be proofread in the language.**
3. The tiltle of manuscript need to improve

**Reply: We have rewritten the title (Page 1, line 1-2).**
4. The existing keywords should be replaced with suitable words, which are not repeated in the title of manuscript

**Reply: The key words have been partially replaced (Page 3, line 47-48).**
5. In introduction section "Especially, non small cell lung cancer (NSCLC) is the most common histology in lung
cancer, accounting for 80%-85% of the total number of lung cancer". Please put the reference if possible

**Reply: We have added (Page 3, line 54).**
6. In discussion section, please improve the discussion section with logics.

**Reply: We have added more detail to the discussion section (Page 12, line 250-255; Page 13, line 261-268; Page 13-14, line 273-281).**
7. in figures 2 B. please recheck about standared errors

**Reply: we have rechecked and revised. Thank you for your comments!**
8. please ensure the style of manuscript according to journal standared.

**Reply: We have revised the manuscript according to the journal's requirements.**

6. PLOS authors have the option to publish the peer review history of their article ([what does this mean?](https://journals.plos.org/plosone/s/editorial-and-peer-review-process#loc-peer-review-history)). If published, this will include your full peer review and any attached files.


If you choose “no”, your identity will remain anonymous but your review may still be made public.


**Do you want your identity to be public for this peer review?** For information about this choice, including consent withdrawal, please see our [Privacy Policy](https://www.plos.org/privacy-policy).

Reviewer #1: No

Reviewer #2: **Yes:** Muhammad Anwar

[NOTE: If reviewer comments were submitted as an attachment file, they will be attached to this email and accessible via the submission site. Please log into your account, locate the manuscript record, and check for the action link "View Attachments". If this link does not appear, there are no attachment files.]

While revising your submission, please upload your figure files to the Preflight Analysis and Conversion Engine (PACE) digital diagnostic tool, <https://pacev2.apexcovantage.com/>. PACE helps ensure that figures meet PLOS requirements. To use PACE, you must first register as a user. Registration is free. Then, login and navigate to the UPLOAD tab, where you will find detailed instructions on how to use the tool. If you encounter any issues or have any questions when using PACE, please email PLOS at [figures@plos.org](mailto:figures@plos.org). Please note that Supporting Information files do not need this step.

**Thanks again to the dear editors and reviewers.**

**Best regards!**
